# Causal relationships between salt intake and gastric cancer: A two-sample Mendelian randomization study

**DOI:** 10.1097/MD.0000000000048510

**Published:** 2026-05-01

**Authors:** Keshuai Li, Jiaqi Wu, Shenglan Lai, Lili Yu, Hua Zhou, Jun Qian, Qibiao Wu

**Affiliations:** a Faculty of Chinese Medicine and State Key Laboratory of Mechanism and Quality of Chinese Medicine, Macau University of Science and Technology, Taipa, Macau, China; b Department of Oncology, Jiangsu Province Hospital of Chinese Medicine, Affiliated Hospital of Nanjing University of Chinese Medicine, Nanjing, Jiangsu Province, China; c Chinese Medicine Guangdong Laboratory (Hengqin Laboratory), Hengqin, China; d Foshan Women and Children Hospital, The Affiliated Foshan Women and Children Hospital, Guangdong Medical University, Foshan, Guangdong, China; e State Key Laboratory of Traditional Chinese Medicine Syndrome, Guangdong Provincial Hospital of Chinese Medicine, Guangdong Provincial Academy of Chinese Medical Sciences, The Second Affiliated Hospital of Guangzhou University of Chinese Medicine, Guangzhou, China.

**Keywords:** causal inference, gastric cancer, genome-wide association study (GWAS), Mendelian randomization, salt intake

## Abstract

The link between salt intake and the risk of gastric cancer remains uncertain, and a causal relationship has not been established. Therefore, clarifying the causal effect of salt intake on the risk of gastric cancer using reliable causal inference methods is necessary. The aim of this study was to assess the causal association between salt intake and cancer by integrating summary-level genome-wide association study (GWAS) data. Two-sample Mendelian randomization (MR) analyses were performed using summary statistics from a GWAS. Inverse-variance weighted (IVW) regression, Mendelian randomization-Egger (MR–Egger) regression, and weighted median analyses were used to evaluate the causal relationship between salt intake and gastric cancer. Moreover, Mendelian Randomization Pleiotropy RESidual Sum of Squares and Outliers and MR–Egger analyses were used to evaluate the level of multipotency, and “leave-one-out” sensitivity analysis was assessed. The IVW method estimate indicated that salt intake was not correlated with gastric cancer incidence. The IVW (β = 0.1008, standard error [SE] = 0.1510, odds ratio [OR] = 1.1061, 95% confidence interval [CI], 0.82–1.48, *P* = .5042), MR–Egger regression (β = 0.059, SE = 0.5318, OR = 1.0612, 95% CI, 0.37–3.01, *P* = .9111), and weighted median (β = 0.2639, SE = 0.2298, OR = 1.3020, 95% CI, 0.82–2.04, *P* = .2508) analyses revealed no causal relationship between salt intake and gastric cancer risk (*P* > .05). In addition, the funnel plot and MR–Egger analysis (*P* = .6694 > .05) did not indicate horizontal pleiotropy or heterogeneity. GWAS data from public databases were used in this study, and the causal relationship between salt intake and gastric cancer risk was analyzed via a two-sample MR method. The results revealed no genetic causal relationship between salt intake and gastric cancer.

## 1. Introduction

Gastric cancer is a type of digestive system tumor, and its development results from the combined influence of external environmental and internal factors. According to global cancer statistics, in 2022, more than 968,000 new cases of gastric cancer and nearly 660,000 deaths were reported. Although its incidence varies across different geographies and ethnic groups, it remains one of the most common cancers and the leading cause of cancer-related deaths.^[1,2]^ This disease continues to pose a significant public health burden and is, at least in part, a preventable public health problem worldwide. Both the incidence and mortality rates rank fifth overall.^[2]^ Currently, *Helicobacter pylori (H pylori*) infection is recognized as a risk factor for the development of gastric cancer.^[3]^ Evidence-based medicine has also revealed that factors such as smoking, alcohol consumption, and high salt intake contribute to the risk of gastric cancer. However, no practical or effective measures for preventing gastric cancer currently exist. Therefore, to effectively prevent gastric cancer, the risk factors associated with its onset must be further clarified.

Sodium is the most abundant electrolyte in extracellular fluid and plays important roles in regulating fluid volume, plasma osmotic pressure, and cell membrane potential. Although salt is a common seasoning in daily life, excessive intake of unhealthy dietary salt has been recognized as a major health hazard worldwide. Advanced technologies can reduce salt use in food, but excessive dietary salt intake remains common. Gastric cancer is a prevalent tumor, and dietary factors, including salt intake, are believed to be associated with its onset. *H pylori* infection is a known risk factor for gastric cancer, and excessive salt intake may promote its colonization in the stomach.^[4,5]^ Evidence indicates that gastric cancer is strongly associated with certain dietary factors, such as salt content and the habitual consumption of salt-rich foods. The high levels of nitrate and nitrite commonly found in these foods promote the formation of carcinogenic N-nitroso compounds. High salt intake may also be harmful to the gastric mucosa, leading to chronic inflammation and glandular atrophy, increased DNA damage and cell proliferation, and increased susceptibility to *H pylori* infection.^[6]^

However, the results of epidemiological studies on the causal relationship between salt intake and gastric cancer are inconsistent. A study was conducted in Poland to estimate dietary salt intake on the basis of weekly salt intake frequency from food, revealing that adding salt to food had minimal effect on gastric cancer incidence.^[7]^ A meta-analysis revealed that higher salt intake increases the risk of developing gastric cancer.^[8]^ Given that conventional observational studies are susceptible to confounding and reverse causation, a causal relationship cannot be established on the basis of this conflicting evidence. Notably, Mendelian randomization (MR) studies, which are less prone to such biases, have begun to provide insights into this issue. Therefore, clarifying the causal effect of salt intake on gastric cancer risk using reliable causal inference methods is necessary.

MR involves the use of single-nucleotide polymorphisms (SNPs) as instrumental variables (IVs) to establish causal associations between exposure and outcome. Because genetic variation is randomly assigned during gamete formation and is largely unaffected by environmental or lifestyle factors, MR can overcome the influence of confounding factors and reverse causality in causal inference. The method leverages the natural randomization of genetic alleles during meiosis, which approximates a randomized experiment under the core assumptions of MR: the genetic instruments are robustly associated with the exposure; they affect the outcome only through the exposure; and they are not confounded by other factors. By exploiting the fixed nature of germline genetic variation, MR mitigates biases from reverse causation and residual confounding often present in conventional observational studies.

In this study, we applied a two-sample MR design to investigate the potential causal effect of salt intake on gastric cancer risk. This approach utilizes summary-level genetic association data from large-scale, independent genome-wide association studies (GWASs) for the exposure (salt intake) and the outcome (gastric cancer), enhancing statistical power and generalizability while maintaining methodological rigor.

## 2. Materials and methods

### 2.1. Study selection and data extraction

**Figure 1.**
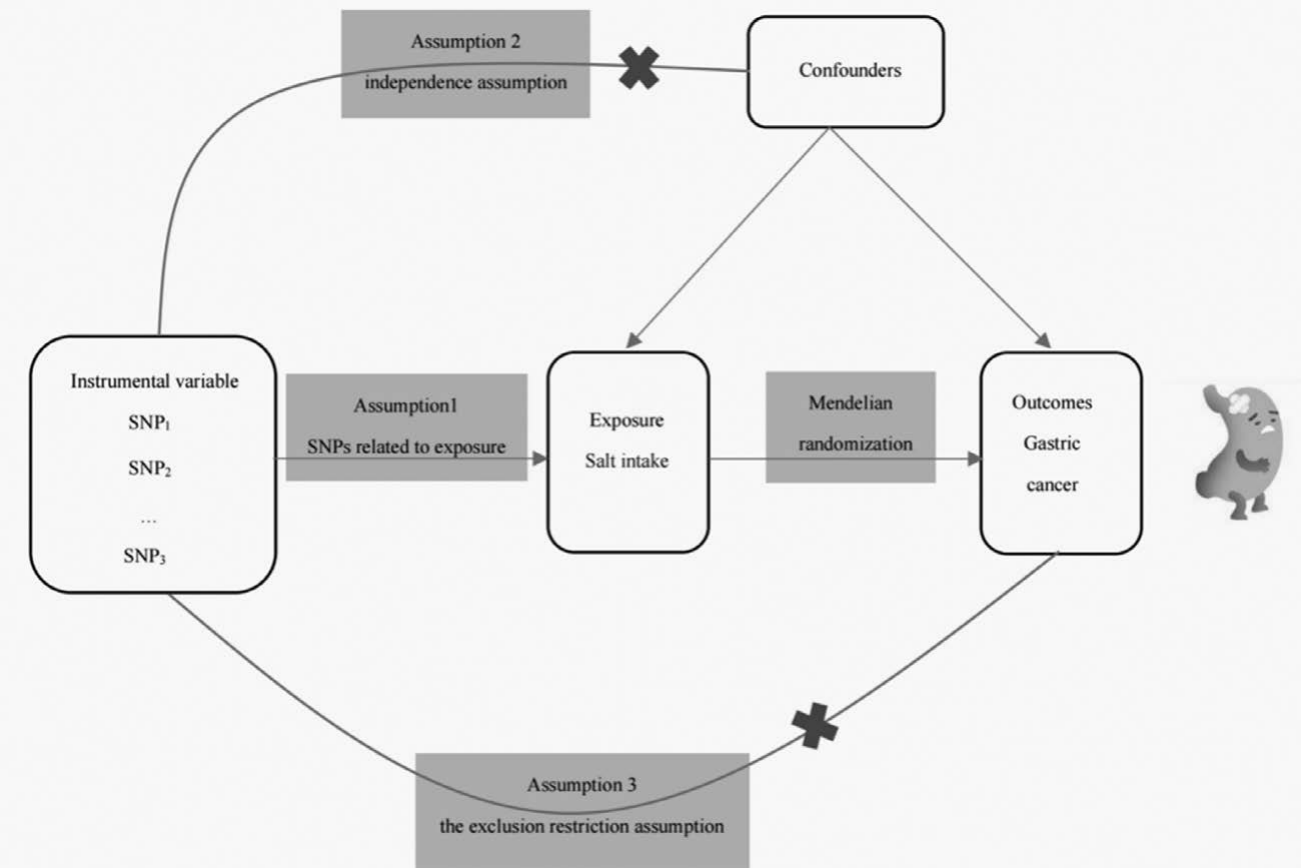
Two-sample Mendelian randomization model. SNP = single-nucleotide polymorphism.

This study is reported according to the Strengthening the Reporting of Observational Studies in Epidemiology using Mendelian randomization guidelines.^[9]^ The overall study design is illustrated in Figure 1. The GWAS data for both salt intake and gastric cancer were obtained from https://gwas.mrcieu.ac.uk. Pooled data on dietary salt intake included up to 462,630 participants of European ancestry. All dietary data were assessed as categorical variables via a questionnaire. The survey on dietary salt intake included the following question: “Do you add salt to your food? (not including salt used in cooking).” The alternative responses were as follows: “Never/rarely,” “usually,” “sometimes,” “always,” and “prefer not to answer” (https://biobank.ctsu.ox.ac.uk/crystal/field.cgi?id=1478). Pooled data on gastric cancer were obtained from the GWAS ebi-a dataset, which included 476,116 individuals of European ancestry (ncase = 1029; ncontrol = 475,087). Because of the use of different data sources, the samples did not overlap. The specific data and information for this study are shown in Table 1.

**Table 1:** Summary of the GWAS included in this two-sample MR study.

	GWAS ID	Trait	Consortium	Sample size	Number of SNPs	Population
Salt intake	ukb-b-8121	Salt added to food	MRC-IEU	462,630	9,851,867	European
Gastric cancer	ebi-a-GCST90018849	Gastric cancer	NA	476,116	24,188,662	European

GWAS = genome-wide association study, MR = Mendelian randomization, MRC-IEU = Medical Research Council Integrative Epidemiology Unit, SNPs = single-nucleotide polymorphisms.

MR requires that the selected SNPs are strongly correlated with the added salt in food. To ensure a strong correlation among the selected SNPs and to avoid bias caused by weak IVs, we set *F* > 10 to indicate no weak instrumental bias. The calculation formula is


$$\begin{array}{l} F=\frac{N-K-1}{K}\times \frac{R^{2}}{1-R^{2}}, \end{array}$$

where *N* is the sample size in the exposure database, *K* is the sample size, and *R*^2^ is the proportion of variation explained by SNPs in the exposure database.^[10]^ The calculation formula is


$$\begin{array}{l} R^{2}=2\times \left(1-MAF\right)\times MAF\times {\left(\frac{\beta }{SD}\right)}^{2}, \end{array}$$

where MAF is the frequency of secondary alleles, β is the allele effect value, and SD is the standard deviation.

### 2.2. MR analysis

In this study, two-sample MR was used to evaluate the causal relationship between adding salt to food and gastric cancer. Three assumptions are necessary for MR studies to guarantee the validity of the results^[11]^: SNPs are strongly correlated with exposure (salt intake; i.e., the hypothesis of association); SNPs are not correlated with confounding factors (i.e., the assumption of independence); and SNPs influence outcomes (gastric cancer) only through exposure (i.e., the exclusion restriction assumption). In this study, the inverse-variance weighted (IVW) method was used as the primary analysis method. Although the IVW method has strong causal detection ability, it relies on the assumption that genetic variation affects outcomes solely through exposure. Although known SNPs associated with confounding were excluded as much as possible in this study, many unknown confounding factors may still bias the results. Therefore, 2 other methods, Mendelian randomization-Egger (MR–Egger) regression and weighted median analyses, were also used for MR analysis. If all 3 MR methods yield similar results for causal inference, these results are considered reliable.

### 2.3. Sensitivity analyses

Cochran *Q* was used to assess heterogeneity among SNPs.^[12]^ If no significant heterogeneity was observed (*P* > .05), a fixed-effects model was applied; otherwise, a random-effects model was used. Various methods were used to evaluate pleiotropy: the MR–Egger intercept as an indicator of directional pleiotropy^[13]^; the “leave-one-out” method for sensitivity analysis of the results to determine the degree of impact of individual SNPs on causal relationships; and the Mendelian Randomization Pleiotropy RESidual Sum of Squares and Outliers method as an indicator for evaluating and correcting level pleiotropy.^[14]^

All statistical analyses were performed using the R packages MR (version 0.4.3; University of Cambridge, Cambridge, UK) and TwoSampleMR package (version 0.5.7; MRC Integrative Epidemiology Unit, Bristol, UK) with R software (version 4.3.2; R Foundation for Statistical Computing, Vienna, Austria). *P* < .05 was considered statistically significant for both sides.

## 3. Results

### 3.1. Genetic instrument selection

In the present study, the harmonization of effect alleles between the exposure and outcome datasets was performed automatically using the TwoSampleMR R package (which was used for the primary analysis), following its standard protocol (Supplementary Documents 1: Supplementary Code, Supplemental Digital Content, https://links.lww.com/MD/R774). GWAS data and screened SNP loci with genome-wide significance (*P* < 5 × 10^−8^) were included for pooled aggregation. Additionally, to prevent linkage disequilibrium from affecting the results, a clustering process was performed by setting the parameter (*R*^2^) threshold (*R*^2^ < 0.001 and region width = 10,000 kb) to assess linkage disequilibrium among the SNPs and ensure independence. To further reduce horizontal pleiotropy, we searched the PhenoScanner database for associated phenotypes for each SNP, and rs7982263 was excluded because of its correlation with potential risk factors for gastric cancer. The final 101 SNPs were selected as IVs for salt intake. All SNPs were strongly correlated with gastric cancer risk (*F* > 10), indicating no weak IV bias and supporting the reliability of the results. The specific data for this study are shown in Table 2.

**Table 2:** Genetic instrument selection for salt intake and gastric cancer.

	SNP	effect_allele.exposure	other_allele.exposure	effect_allele.outcome	other_allele.outcome	*R* ^2^	FSTAT
1	rs1008078	T	C	T	C	9.00390142921487e−05	41.658319978016
2	rs10128297	T	C	T	C	7.43138288054713e−05	34.3822130665134
3	rs10140751	T	G	T	G	6.55853667352143e−05	30.3436171392369
4	rs1045411	T	C	T	C	0.000123562165842676	57.1703817556574
5	rs10736951	C	G	C	G	8.2152352811982e−05	38.0091012137946
6	rs10752999	C	A	C	A	9.47832652079707e−05	43.8535489991982
7	rs10883796	A	G	A	G	7.5615399346394e−05	34.9844463317038
8	rs10971930	C	T	C	T	7.43155431566896e−05	34.3830062912809
9	rs11022746	G	T	G	T	8.25973527480234e−05	38.2150045653249
10	rs11075194	G	A	G	A	6.51933530910171e−05	30.1622369311533
11	rs11082431	T	C	T	C	6.53928395984645e−05	30.2545370278454
12	rs11126666	A	G	A	G	7.61925109431343e−05	35.2514748509837
13	rs11210985	A	G	A	G	0.000160276384263047	74.1602292322426
14	rs11761254	T	C	T	C	0.000104240564160407	48.2296312003665
15	rs12094804	G	A	G	A	6.80035935112306e−05	31.462506022384
16	rs12501838	T	A	T	A	0.000107304880015046	49.6475694580844
17	rs12563932	C	A	C	A	6.46461493967896e−05	29.9090523082018
18	rs12579997	C	G	C	G	0.000100764695410019	46.6212672859442
19	rs12658060	C	T	C	T	0.000362137504095227	167.595641912026
20	rs12789951	T	C	T	C	9.19012996685986e−05	42.5200221083781
21	rs12988960	C	G	C	G	0.00013486581049041	62.4011159526361
22	rs13131880	C	T	C	T	7.60425039853478e−05	35.1820668661934
23	rs13400612	G	C	G	C	0.000213144382676329	98.6275813837336
24	rs1726866	A	G	A	G	0.000485893778472199	224.897343166876
25	rs1728779	G	A	G	A	6.57843826293591e−05	30.4356995607606
26	rs17805497	C	T	C	T	9.83634561689478e−05	45.5101655377011
27	rs1823011	G	A	G	A	6.52431455535797e−05	30.185275323473
28	rs1895951	C	T	C	T	6.43797434295199e−05	29.7857895448007
29	rs2263636	C	A	C	A	7.09522833705832e−05	32.8268420905684
30	rs2339234	A	G	A	G	0.000186301975020304	86.2045701813738
31	rs2457427	C	T	C	T	9.00155516358472e−05	41.6474635415937
32	rs2463710	A	T	A	T	6.43103944977496e−05	29.7537026580606
33	rs2506738	G	A	G	A	0.000152007967114413	70.3338331131974
34	rs2521501	T	A	T	A	7.77152290358537e−05	35.9560353099183
35	rs2547040	C	G	C	G	7.02278338598093e−05	32.4916441406824
36	rs264932	G	A	G	A	7.22850941301089e−05	33.4435259956487
37	rs2693687	T	C	T	C	8.81786477188647e−05	40.7975089060486
38	rs2736748	G	A	G	A	0.000363546340657158	168.247882387505
39	rs2835623	T	C	T	C	6.47367936359993e−05	29.9509922974426
40	rs28366169	A	G	A	G	8.62935140836655e−05	39.9252411228551
41	rs2852348	G	A	G	A	0.000117704727498347	54.4599128623082
42	rs2899345	C	T	C	T	6.72076556102117e−05	31.0942330701487
43	rs324018	G	T	G	T	6.59946808176568e−05	30.5330022130464
44	rs329670	C	T	C	T	7.29147642099329e−05	33.7348713070998
45	rs33137	T	C	T	C	0.000100379702462225	46.4431229375563
46	rs34906832	G	A	G	A	7.69632746215542e−05	35.6081063280865
47	rs35099536	C	A	C	A	0.000141559813508152	65.4988054023272
48	rs35142265	G	A	G	A	0.000106739464330652	49.3859363327505
49	rs35271178	T	C	T	C	7.07159552375549e−05	32.7174945885223
50	rs35702851	C	G	C	G	6.7000528153619e−05	30.99839725
51	rs3890316	A	G	A	G	6.65944557317317e−05	30.8105116755154
52	rs400750	G	T	G	T	0.000183293950713728	84.8124594415513
53	rs4235642	G	A	G	A	9.89981758868159e−05	45.8038626130131
54	rs4236065	C	T	C	T	6.54196379578922e−05	30.26693632
55	rs429358	C	T	C	T	8.59322906730139e−05	39.7581002741044
56	rs4595499	T	C	T	C	0.000197412722076263	91.3466857865911
57	rs4739105	C	T	C	T	0.000108753391130178	50.3178360670788
58	rs4799949	T	C	T	C	7.43517437157768e−05	34.3997561735973
59	rs4860797	A	G	A	G	9.00381199751406e−05	41.6579061674128
60	rs4912891	C	T	C	T	6.63607161437565e−05	30.7023628189378
61	rs491907	G	A	G	A	8.39358986288333e−05	38.834356507471
62	rs4948275	T	C	T	C	0.000111192969520588	51.4467016148953
63	rs4981196	C	A	C	A	8.54037943091312e−05	39.5135611616946
64	rs526210	A	G	A	G	6.65921479849136e−05	30.8094439050125
65	rs528301	A	G	A	G	0.000160646377811353	74.3314535528815
66	rs55897719	A	C	A	C	0.000109608586562077	50.7135598256524
67	rs586716	A	G	A	G	0.000138711235060262	64.1806038242824
68	rs62098445	A	C	A	C	0.000148865425794161	68.8795679905185
69	rs6416794	C	T	C	T	6.42841761548911e−05	29.741571758623
70	rs6443950	T	A	T	A	0.000130137323844218	60.2130058148404
71	rs667128	T	C	T	C	7.31095675822827e−05	33.8250059630157
72	rs6695915	G	A	G	A	6.64837324022764e−05	30.7592811456169
73	rs6776248	C	T	C	T	6.96399518630258e−05	32.2196354243479
74	rs6780346	T	C	T	C	0.000185524296935171	85.8446607128487
75	rs6804929	A	G	A	G	7.22626970867962e−05	33.4331629984007
76	rs6887291	G	T	G	T	0.000102964675981842	47.639247279788
77	rs6987313	C	T	C	T	6.80363686683711e−05	31.4776707907259
78	rs7021360	A	C	A	C	6.75496463279086e−05	31.2524688746071
79	rs7110845	G	A	G	A	9.01703579025129e−05	41.7190941613756
80	rs72807804	T	C	T	C	6.87556979977404e−05	31.8104982063065
81	rs73040343	G	A	G	A	7.03631847694735e−05	32.5542700656516
82	rs736935	T	C	T	C	0.000150698208613608	69.7277186967946
83	rs7465705	A	G	A	G	9.20029826043084e−05	42.567072133863
84	rs7581335	T	A	T	A	7.50895126777309e−05	34.7411197648263
85	rs7591518	C	T	C	T	0.000250386565531467	115.864847039808
86	rs7670308	A	G	A	G	9.02572055709847e−05	41.7592795747746
87	rs7673170	A	G	A	G	0.000105693264652136	48.9018322337848
88	rs7927679	T	C	T	C	0.000155317495870559	71.8653844311547
89	rs8022455	C	T	C	T	8.52569449261777e−05	39.4456129297569
90	rs8040685	T	C	T	C	6.74444326602453e−05	31.2037875144897
91	rs8097544	G	A	G	A	6.73833000861193e−05	31.1755020604519
92	rs868720	C	G	C	G	0.00014924082591171	69.0532903799709
93	rs9278020	A	G	A	G	0.000142337906315501	65.8588751373268
94	rs9317406	T	C	T	C	7.44131567783149e−05	34.4281718029842
95	rs9569747	G	T	G	T	9.10058145315388e−05	42.1056698258757
96	rs961044	T	C	T	C	6.63999496108772e−05	30.7205157292774
97	rs9611875	G	A	G	A	0.0004637316681012	214.634787098176
98	rs9667150	A	G	A	G	0.000108036129083445	49.9859386129322
99	rs9835772	T	A	T	A	6.73006381298022e−05	31.1372551738569
100	rs9843358	T	C	T	C	0.000141272809065984	65.3659915498219
101	rs99780	T	C	T	C	7.53269319598171e−05	34.8509730955854

SNP = single-nucleotide polymorphism.

### 3.2. MR analysis

**Figure 2.**
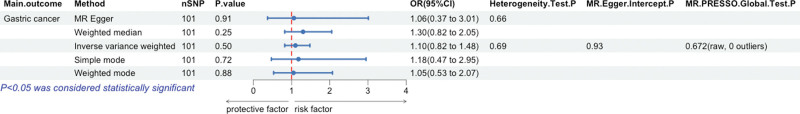
Forest plot of the MR study. CI = confidence interval, OR = odds ratio, SNP = single-nucleotide polymorphism.

**Figure 3.**
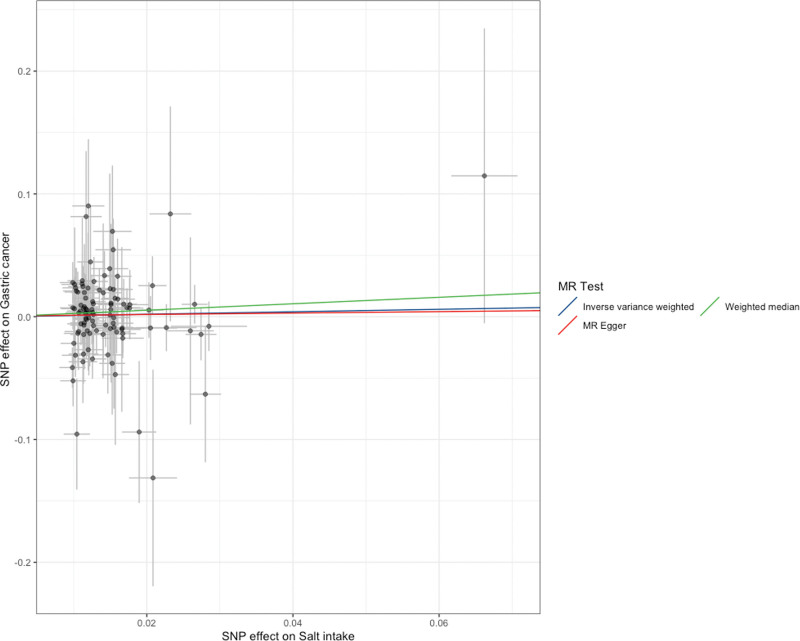
Scatter plot. MR = Mendelian randomization, SNP = single-nucleotide polymorphism.

**Figure 4.**
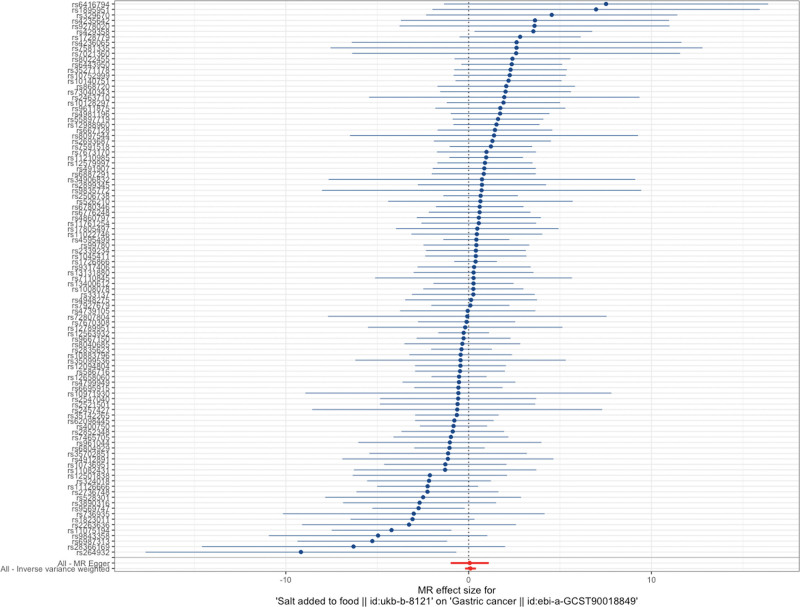
Forest plot. MR = Mendelian randomization.

The MR analysis results indicate that the IVW method does not support a causal relationship between salt intake and gastric cancer (standard error [SE] = 0.15106, *P* = .504 > .05; Figs. 2 and 3). MR–Egger analysis also revealed no causal relationship between salt intake and gastric cancer (SE = 0.531, *P* = .91118; Figs. 2 and 4). Similarly, the weighted median method also did not reveal a causal relationship between salt intake and gastric cancer (SE = 0.22987, *P* = .25087; Figs. 3 and 4). The MR values determined via the IVW, MR–Egger, and weighted median methods are consistent. Therefore, the MR analysis results indicate that there is no causal relationship between salt intake and gastric cancer.

### 3.3. Sensitivity analyses

**Figure 5.**
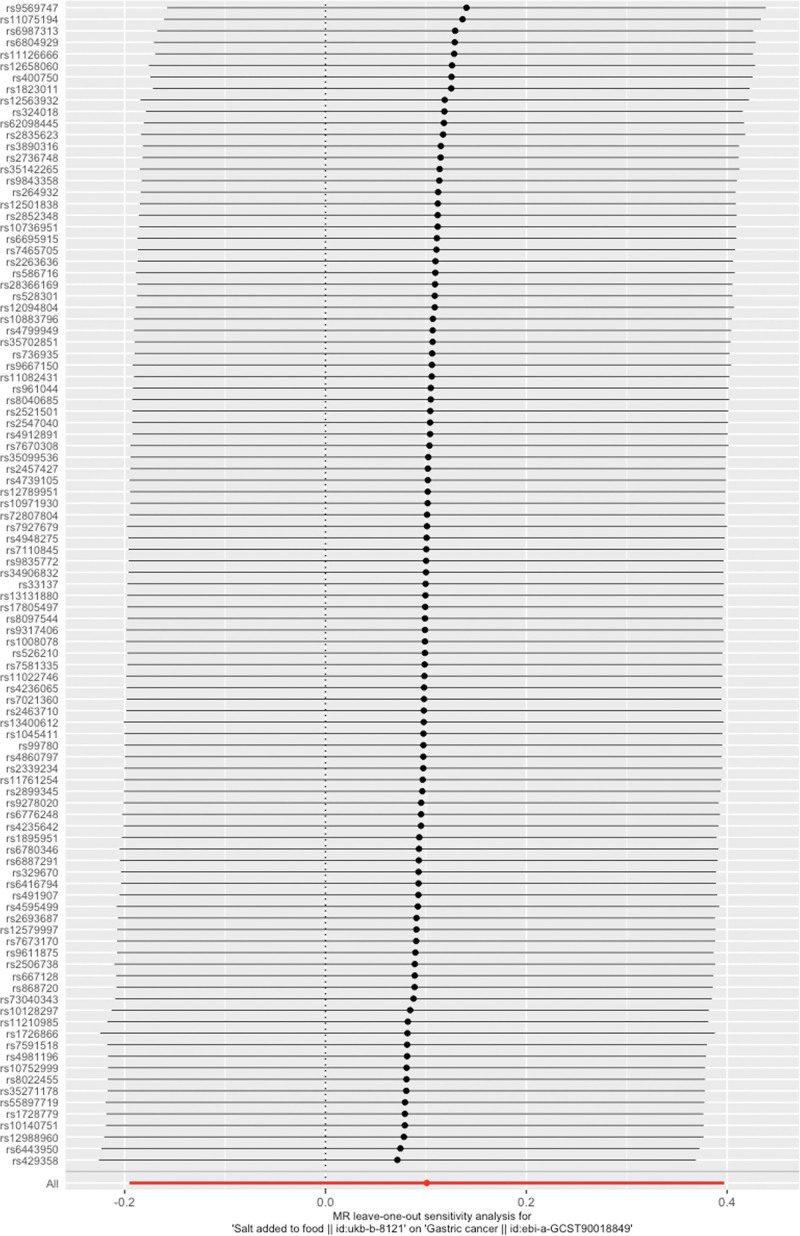
MR leave-one-out sensitivity analyses. MR = Mendelian randomization.

**Figure 6.**
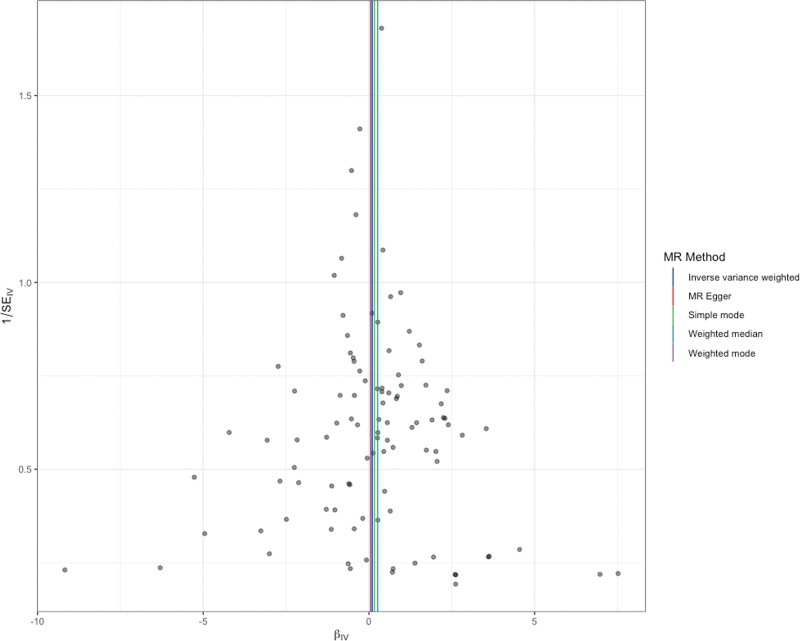
Funnel plot. IV = instrumental variable, MR = Mendelian randomization, SE = standard error.

The Cochran *Q* values for the IVW (*P* = .694976 > .05) and MR–Egger regression (*P* = .66949 > .05) methods both indicated no heterogeneity among the SNPs (Fig. 2). Therefore, a fixed-effects model was used in this study. The intercept of the MR–Egger regression method was close to 0 (*P* = .93545 > .05; Fig. 4), indicating no evidence of directional pleiotropy. The leave-one-out analysis revealed that after each SNP was sequentially removed, the IVW analysis results for the remaining 100 SNPs were similar to those obtained when all SNPs were included. No SNPs with a significant impact on causal relationships were identified, as shown in Figure 5. The Mendelian Randomization Pleiotropy RESidual Sum of Squares and Outliers analysis revealed no significant outliers among the SNPs included in this study. Additionally, the funnel and density plots (Fig. 6) indicate that causal relationships are less likely to be affected by potential biases.

## 4. Discussion

In this study, GWAS data from public databases were used, and the causal relationship between salt intake and gastric cancer risk was analyzed using a two-sample MR method. The results indicate no causal relationship between salt intake and gastric cancer.

High-salt diets can accelerate the development of gastric cancer.^[15]^ *H pylori* is recognized as the strongest risk factor for the development of gastric cancer.^[16]^ Elevated salt levels upregulate cagA expression in certain *H pylori* strains, augmenting its translocation to gastric epithelial cells and thereby potentiating the carcinogenicity of cagA-positive strains.^[17]^ High salt concentrations in the stomach, acting as a gastric mucosal irritant, cause damage to the mucosal barrier, leading to gastric epithelial hyperplasia,^[8]^ and increase the likelihood of endogenous mutations leading to glandular atrophy and DNA damage, which provide a basis for the development of gastric cancer.^[18]^ High salt intake introduces elevated nitrate/nitrite levels,^[19]^ which may form N-nitroso compounds and enhance their carcinogenicity, while also promoting the activity of other carcinogens.^[20]^ High salt intake impairs gastric mucosal defense and immune balance, promoting *H pylori* infection and precancerous transformation.^[21]^ High salt intake accelerates intestinal metaplasia via elevated gastric NaCl, increasing gastric cancer susceptibility.^[22]^ While these mechanisms are biologically plausible, our genetic findings do not provide supporting evidence for their causal role.

The present MR study is the first in which the causal relationship between salt intake and gastric cancer risk is comprehensively evaluated. The GWAS database was used to identify 101 SNPs, and 3 different models were applied to determine their causal relationships. However, MR analysis results can be influenced by pleiotropy.^[23]^ Therefore, weighted median analysis was used to eliminate pleiotropy, yielding effective MR analysis results even if 50% of the SNPs were not effective IVs. MR–Egger regression was also used to evaluate the causal effects of imbalanced pleiotropy testing and the effect of the exposure on the outcomes.^[24]^ The intercept *P* value indicated that the MR–Egger method did not yield evidence of imbalanced pleiotropy. The study data do not support a causal relationship between salt intake and gastric cancer risk. The previously reported association may be influenced by bias or confounding factors in observational studies.

This study has several limitations. First, all the GWAS data were derived from European populations, and additional research is needed to determine if the study findings are generalizable to other ethnic groups. Second, salt intake was analyzed as a whole in the present study, without stratification by salt type, consumption pattern, intake level, or dietary habits across different regions. Additionally, the use of a self-reported, categorical salt preference variable may have limited our ability to detect a true causal effect, as measurement error and potential misclassification could attenuate genetic associations. This methodological constraint should be considered when interpreting our null findings.

Furthermore, the authors explicitly acknowledge that the genetic instruments and outcome data used in this study are exclusively derived from individuals of European ancestry. This presents a significant limitation in the context of gastric cancer, as the disease burden is disproportionately higher in East Asian populations.^[2]^ Notably, East Asian populations have distinct genetic backgrounds and dietary profiles, including salt consumption habits, which may differ substantially from those in European cohorts. Therefore, although the study findings offer insights into the European context, they cannot be generalized to Asian or other non-European populations. Future MR studies using large-scale GWASs from East Asian populations and other diverse groups are essential to validate or refute these associations and explore potential unique gene–environment interactions in high-risk regions.

In summary, in this study, MR was used to comprehensively evaluate whether salt intake increases cancer risk. The findings revealed no evidence of a causal genetic association between salt intake and gastric cancer risk in European populations.

## Author contributions

**Conceptualization:** Keshuai Li.

**Funding acquisition:** Qibiao Wu, Jun Qian.

**Methodology:** Keshuai Li, Lili Yu.

**Formal analysis:** Keshuai Li, Jiaqi Wu.

**Project administration:** Qibiao Wu, Hua Zhou.

**Supervision:** Qibiao Wu, Lili Yu, Hua Zhou, Jun Qian.

**Data curation:** Shenglan Lai.

**Validation:** Shenglan Lai.

**Writing – original draft:** Keshuai Li, Jiaqi Wu.

**Writing – review & editing:** Keshuai Li, Jiaqi Wu.

## Supplementary Material
