# Supplementary material for: Causal relationships between salt intake and gastric cancer: A two-sample Mendelian randomization study
Source: Medicine (Baltimore). 2026 May 1;105(18):e48510. doi: 10.1097/MD.0000000000048510 (PMC13138452; doi:10.1097/MD.0000000000048510)
Supplement: Supplementary file 1 [file medi-105-e48510-s001.pdf]

Supplementary code

```
library(TwoSampleMR)
library(ieugwasr)
library(MRPRESSO)
library(ggsci)
library(ggplot2)
library(grid)
library(forestploter)
Sys.setenv(OPENGWAS_JWT="eyJhbGciOiJSUzI1NiIsImtpZCI6ImFwaS1qd3QiLCJ0eXAiOiJ
KV1QifQ.eyJpc3MiOiJhcGkub3Blbmd3YXMuaW8iLCJhdWQiOiJhcGkub3Blbmd3YXMuaW8i
LCJzdWIiOiJzeWp5MTg2ODQzODA2ODhAb3V0bG9vay5jb20iLCJpYXQiOjE3MjQyMTg2M
zMsImV4cCI6MTcyNTQyODIzM30.wpc4m5dNRkSElKpcUxUHPJDwJydRjEGPYPaKC-JaYW
OQPzRAmaOs93epoZxV8DLBrx8gyZqfI7OdtQhkEuPAyvw5P2Wx8SJmpVUBbRD6tC1ITtaR0
aHg7dmBY7ab4QMmTVjw8je9P9rctY9TfOuRIwFhKN2PnyQvEfXCMeZnDsh8-y3iMLkm8_G
pH4JntA88q8xurOlZRYR2GHrKaijEQdB1pfC9xFQVCeqsjrO0HM6UkQF61mQL1DqEppxW4
CJuxkGZLmzDJal0JC_4j6urMkBWlFO96lsOOxgi_ON5U5gOlMOBptXtx1RiYws_oJWYRVs_c
whBJPPuBZJeGRsG1w")


exp <- extract_instruments(outcomes = 'ukb-b-8121',p1 = 5e-08,clump = TRUE,r2 = 0.001,kb = 10000)
colnames(exp)
write.csv(exp,'exp.csv')
out<-extract_outcome_data(snps = exp$SNP,outcomes = 'ebi-a-GCST90018849', proxies=TRUE)
write.csv(out,'out.csv')
dat<-harmonise_data(exp,out,action = 2)
Harmonising Salt added to food || id:ukb-b-8121 (ukb-b-8121) and Gastric cancer || id:ebi-a-GCST90018849 (ebi-a-GCST90018849)
Removing the following SNPs for incompatible alleles:
rs55897719
Removing the following SNPs for being palindromic with intermediate allele frequencies:
rs11130206, rs13084934, rs9375448, rs976179
dat<-dat[dat$mr_keep,]
write.csv(dat,'dat.csv')
phenoscanner removes linkage disequilibrium
dat <- read.csv('dat.csv')
PVEfx <- function(BETA, SE, N){pve <- (BETA^2)/((BETA^2) + ((SE^2)*N)) return(pve) }
dat$PVE <- mapply(PVEfx, dat$beta.exposure, dat$se.exposure, N = dat$samplesize.exposure)
dat$FSTAT <- ((dat$samplesize.exposure - 1 - 1)/1)*(dat$PVE/(1 - dat$PVE))
write.csv(dat,'datF.csv')
results<-mr(dat)
Analysing 'ukb-b-8121' on 'ebi-a-GCST90018849'
results<-mr(dat,method_list = c("mr_ivw","mr_egger_regression","mr_weighted_median"))
```

```
Analysing 'ukb-b-8121' on 'ebi-a-GCST90018849'
write.csv(results,'results.csv')
OR <-generate_odds_ratios(results)
write.csv(OR,'OR.csv')
heterogeneity <- mr_heterogeneity(dat)
write.csv(heterogeneity,'heterogeneity.csv')
pleio <- mr_pleiotropy_test(dat)
write.csv(pleio,'pleio.csv')
mr_presso(BetaOutcome = 'beta.outcome',
+            BetaExposure = 'beta.exposure',
+            SdOutcome = 'se.outcome',
+            SdExposure = 'se.exposure',
+            data = dat, OUTLIERtest = TRUE,
+            DISTORTIONtest = TRUE, SignifThreshold = 0.05, NbDistribution = 1000)
$`Main MR results`
       Exposure       MR Analysis Causal Estimate        Sd      T-stat     P-value
1 beta.exposure               Raw       0.1008887 0.1451507 0.6950617 0.4886286
2 beta.exposure Outlier-corrected              NA        NA        NA        NA

$`MR-PRESSO results`
$`MR-PRESSO results`$`Global Test`
$`MR-PRESSO results`$`Global Test`$RSSobs
[1] 93.92387

$`MR-PRESSO results`$`Global Test`$Pvalue
[1] 0.672



Warning message:
In mr_presso(BetaOutcome = "beta.outcome", BetaExposure = "beta.exposure",  :
  No outlier were identified, therefore the results for the outlier-corrected MR are set to NA
single <- mr_leaveoneout(dat)
> mr_leaveoneout_plot(single)
$`ukb-b-8121.ebi-a-GCST90018849`

attr(,"split_type")
[1] "data.frame"
attr(,"split_labels")
  id.exposure          id.outcome
1  ukb-b-8121 ebi-a-GCST90018849
Warning messages:
1: Removed 1 row containing missing values or values outside the scale range
(`geom_errorbarh()`).
```

```
2: Removed 1 row containing missing values or values outside the scale range (`geom_point()`).
> dev.off()
scatter_plot<-mr_scatter_plot(results,dat)[[1]]+scale_color_lancet()+scale_fill_lancet()+theme_bw()
Scale for colour is already present.
Adding another scale for colour, which will replace the existing scale.
scatter_plot[["layers"]][[3]][["aes_params"]]$colour<-"black"
scatter_plot[["layers"]][[3]][["aes_params"]]$alpha<-0.5
scatter_plot[["labels"]][["x"]]<-"SNP effect on Salt intake"
scatter_plot[["labels"]][["y"]]<-"SNP effect on Gastric cancer "
scatter_plot
dev.off()
forest_plot<-mr_forest_plot(mr_singlesnp(dat))[[1]]+
scale_color_lancet()+
scale_fill_lancet()+
theme_bw()+
theme(legend.position = 'none')
Scale for colour is already present.
Adding another scale for colour, which will replace the existing scale.
forest_plot
Warning messages:
1: Removed 1 row containing missing values or values outside the scale range (`geom_errorbarh()`).
2: Removed 1 row containing missing values or values outside the scale range (`geom_point()`).
dev.off()
funnel_plot<-mr_funnel_plot(mr_singlesnp(dat,all_method=c("mr_egger_regression","mr_weighted_median","mr_ivw","mr_simple_mode","mr_weighted_mode")))[[1]]+
+     theme_bw()+
+     scale_color_lancet()+
+     scale_fill_lancet()
Scale for colour is already present.
Adding another scale for colour, which will replace the existing scale.
funnel_plot[["layers"]][[1]][["aes_params"]]$colour <- "black"
funnel_plot[["layers"]][[1]][["aes_params"]]$alpha<-0.5
funnel_plot
dev.off()
density_plot<-mr_density_plot(mr_singlesnp(dat),results)[[1]]+
+     theme_bw()+
+     scale_color_lancet()+
+     scale_fill_lancet()
Scale for colour is already present.
Adding another scale for colour, which will replace the existing scale.
density_plot[["layers"]][[3]][["aes_params"]][["colour"]]<-"black"
density_plot[["layers"]][[3]][["aes_params"]]$alpha<-0.5
```

```
density_plot[["labels"]][["colour"]]<-"Method"
density_plot[["labels"]][["y"]][[1]]<-"Density"
density_plot
dev.off()
dt <- read.csv("OR1.csv",header = T,fileEncoding = 'GBK')
dt$` ` <- paste(rep(" ", 30), collapse = " ")
dt$'OR(95%CI)'<-ifelse(is.na(dt$or),"",
+                                     sprintf('%.2f(%.2f to %.2f)',
+                                                 dt$or,dt$or_lci95,dt$or_uci95))
dt[is.na(dt)] <- " "
tm <- forest_theme(base_size = 10,
+                               ci_pch = 20,
+                               ci_col = "#4575b4", # #762a83
+                               ci_lty = 1,
+                               ci_lwd = 2.3,
+                               ci_Theight = 0.2,
+                               refline_lwd = 1.5,
+                               refline_lty = "dashed",
+                               refline_col = "red",
+                               summary_fill = "#4575b4",
+                               summary_col = "#4575b4",
+                               footnote_cex = 1.1,
+                               footnote_fontface = "italic",
+                               footnote_col = "blue")
p <- forest(dt[,c(1:4, 11:12,8:10)],
+                     est = dt$or,#HR
+                     lower = dt$or_lci95,
+                     upper = dt$or_uci95,
+                     sizes = 0.6,
+                     ci_column = 5,
+                     ref_line = 1,
+                     xlim = c(0,4),
+                     ticks_at = c(0,1,2,3,4),
+                     arrow_lab = c('protective factor','risk factor'),
+                     footnote = 'P<0.05 was considered statistically significant',
+                     theme = tm)
print(p)
png(filename = 'forest2.tif',width = 8000,height = 2800,res = 350)
print(p)
dev.off()
```
